# Pair Housing of Dairy Calves and Age at Pairing: Effects on Weaning Stress, Health, Production and Social Networks

**DOI:** 10.1371/journal.pone.0166926

**Published:** 2017-01-04

**Authors:** Sarah L. Bolt, Natasha K. Boyland, David T. Mlynski, Richard James, Darren P. Croft

**Affiliations:** 1 Centre for Research in Animal Behaviour, College of Life and Environmental Sciences, University of Exeter, Exeter, United Kingdom; 2 Agriculture and Horticulture Development Board, Kenilworth, Warwickshire, United Kingdom; 3 Compassion in World Farming, River Court, Mill lane, Godalming, Surrey, United Kingdom; 4 Department of Physics, Centre for Networks and Collective Behaviour, University of Bath, Bath, United Kingdom; University of British Columbia, CANADA

## Abstract

The early social environment can influence the health and behaviour of animals, with effects lasting into adulthood. In Europe, around 60% of dairy calves are reared individually during their first eight weeks of life, while others may be housed in pairs or small groups. This study assessed the effects of varying degrees of social contact on weaning stress, health and production during pen rearing, and on the social networks that calves later formed when grouped. Forty female Holstein-Friesian calves were allocated to one of three treatments: individually housed (*I*, n = 8), pair-housed from day five (*P5*, n = 8 pairs), and pair-housed from day 28 (*P28*, n = 8 pairs). From day 48, calves were weaned by gradual reduction of milk over three days, and vocalisations were recorded as a measure of stress for three days before, during and after weaning. Health and production (growth rate and concentrate intakes) were not affected by treatment during the weaning period or over the whole study. Vocalisations were highest post-weaning, and were significantly higher in *I* calves than pair-reared calves. Furthermore, *P28* calves vocalised significantly more than *P5* calves. The social network of calves was measured for one month after all calves were grouped in a barn, using association data from spatial proximity loggers. We tested for week-week stability, social differentiation and assortment in the calf network. Additionally, we tested for treatment differences in: coefficient of variation (CV) in association strength, percentage of time spent with ex-penmate (*P5* and *P28* calves only) and weighted degree centrality (the sum of the strength of an individual’s associations). The network was relatively stable from weeks one to four and was significantly differentiated, with individuals assorting based on prior familiarity. *P5* calves had significantly higher CV in association strength than *I* calves in week one (indicating more heterogeneous social associations) but there were no significant treatment differences in week four. The mean percentage of time that individuals spent with their ex-penmate after regrouping decreased from weeks 1–4, though treatment did not affect this. There were also no significant differences in weighted degree centrality between calves in each rearing treatment. These results suggest that early pair-rearing can allow calves the stress buffering benefits of social support (and that this is more effective when calves are paired earlier) without compromising health or production, and sheds light on the early development of social behaviour in cattle.

## Introduction

Research shows that early social conditions influence many key factors in an animal’s life, including the development of personality [1], abnormal behaviours [2], stress response [3], susceptibility to infection [4] and wound healing [5]. Environmental effects during early life can even be transmissible to future generations [6]. For many young mammals, the social environment effectively consists of the mother-infant bond, and disrupting this relationship induces a range of biological consequences [7] which can result in persistent changes in neurobiology and behaviour [8]. Such consequences can be seen in a diverse range of taxa (e.g. primates [9], pigs [10], rodents [11]). Individual differences in early social experiences and developmental environment can also lead to consistent individual differences in adult social behaviour [12, 13]. This can be expressed as differences in the way individuals form and maintain social relationships [14], which can affect social network position and overall social group structure [13]. Significant connections between social relationships and biological fitness have emerged in numerous animal studies (see [15]). Therefore understanding how the early social environment affects animals under human management, such as farm animals, is vital for maximising welfare and productivity.

Although there is variation in the way young calves are housed in the dairy industry, an estimated 60% of dairy calves in Europe experience social restriction soon after birth, being reared in individual pens during the milk feeding period [16]. The EU directive on calves (Council Directive 97/2/EC) acknowledges that social contact is important, stating that calves over eight weeks old must be housed in groups. However, for calves under eight weeks old, regulations only stipulate a requirement for visual and tactile contact (e.g. nose-to-nose contact through pen divides) with conspecifics of a similar age. The consequences of restricting social contact during early rearing of calves is not fully understood. Motivations for individual housing of calves include reducing disease transmission and easier detection of health issues [17]. Increased contact between animals can increase the risk of infectious disease spread [18, 19]. A higher incidence of disease in group-housed calves (compared with pair-housed calves) has been reported in some studies (e.g. [20]), however others have demonstrated the opposite result [21, 22], or have found no significant differences in health or disease incidence of calves within each type of rearing system [23, 24].

Early development of social bonds with conspecifics is common in domestic herbivores and preferential bonds between unrelated individuals are often formed, particularly in the absence of the dams [25–28]. Group housing calves may alleviate the stress caused by separation from the dam, via ‘social support’; this term refers generally to a range of benefits provided by social companions that improve an individual’s ability to cope with challenges [29]. Social support has largely been investigated indirectly by measuring the stress-buffering effects of social contact referred to as ‘social buffering’ [29]. Social contact is important to calves, indicated by a willingness to ‘work’ for access to other calves in preference choice tests [30]. In fact, group housing can simulate an age-appropriate social environment: in studies of free-range cattle, calves are observed to spend much of their time resting together in small groups away from their dams [31, 32].

Natural weaning of cattle appears to begin when the calf is around 10 months old [33]. However, on commercial dairy farms, weaning from milk begins as early as five weeks, making this a particularly stressful time for calves [34]. Generally, when cattle are stressed they vocalise more [35]. Increased vocalisation is a common response to weaning and has been used in previous studies as a non-invasive measure of stress [36–38]. When housed in pairs during weaning, calves have been shown to vocalise less and have higher growth rates than calves housed individually [23, 38]. Although there is evidence demonstrating that social companions can buffer stress at weaning, the effect that the strength of the social bond has on social buffering in calves is not fully understood.

In addition to the diet change, dairy calves experience a new physical and social environment following weaning. Calves of a similar age and weight are typically grouped together and moved into new housing facilities, which contain a number of novel items such as feeding and drinking apparatus. For calves previously housed in individual pens, this is the first time they experience full social contact with conspecifics. In contrast, group-reared calves have prior social experience and are likely to have pre-established social bonds with some group members. Rearing method is thus expected to impact stress levels during the process of regrouping. Furthermore, interactions with the physical environment could be affected; indeed there is some evidence that early social conditions impact calves’ exploratory behaviour [39], social facilitation [38], cognition [40, 41] and food neophobia [42].

Not only does early social contact affect calves’ welfare during rearing, it has also been shown to influence behaviour after grouping and into adulthood. Research demonstrates that cattle that were group housed as calves: are more confident [43], show less fear [39], are more cooperative with humans [44], play more [45], are involved in less agonistic encounters [46], and achieve higher social rank [47, 48] than individually housed calves. Additionally, early familiarity between calves is associated with more positive social behaviour later in life. For example, heifers that were reared together were less aggressive and engaged in more non-agonistic interactions (with each other), fed and rested closer together, and were more tolerant in a food-competitive situation, compared to those they were not reared with [49]. Therefore management practices which encourage the development and stability of social bonds are beneficial and should be explored.

The aim of this study was to investigate the effect of the early social environment on the performance and social behaviour of calves. Firstly, we measured the growth, concentrate intake, health and weaning stress (measured by number of vocalisations) of calves in three rearing treatments: individually housed, pair-housed from day five and pair-housed from day 28. Secondly, we measured the social network of the calves over a one-month period when all were grouped together post-weaning (‘barn grouping’), using spatial proximity loggers to measure social associations. We quantified the stability of social relationships in the group and investigated whether the network was socially differentiated (heterogeneous). We investigated whether relationships were affected by prior opportunities to socialise (treatment and familiarity between calves) during pen rearing. The coefficient of variation (CV) in association strength was calculated for each calf, and we tested for treatment differences during week one and four. We explored whether the percentage of time individuals spent with their ex-penmate differed between the pair-housed treatments, and whether it decreased over time after regrouping. Lastly, we tested for differences in social network position between the calves in the three rearing treatments.

## Methods

### Animals, housing and diet

This study was conducted using forty female Holstein-Friesian calves on a commercial farm (with permission to conduct this study from the landowners) in Somerset, UK, from April to July 2013. Calves were separated from their dams after calving and individually housed, until randomly assigned to one of three treatments on day five: individually housed (*I*; n = 8), pair housed from day five (*P5*; n = 8 pairs), or pair housed from day 28 (*P28*; n = 8 pairs). One replicate of each treatment made up a block and there were eight blocks in total (hence total n = 40), with calves born earliest in block one and latest in block eight. As calves were not all born on the same day, a block entered the trial when the mean age of calves was five days. The age difference between the oldest and youngest calves in any one block was (mean ±SD) 2.5 ±1.19 days. All calves had visual access to others via the front opening of pens and some contact to neighbouring pens via four ventilation slots (23cm high, 8.5cm wide) on the pen walls. All pens were bedded with straw, and space per calf (1.22m x 2.13m) was consistent across all treatments. Calves were bucket fed pellets (BOCM, Super Rearer 18 + deccox) from day 4 and water was available *ad libitum* from day one. Milk replacer (150g BOCM Omega Gold per litre of warm water) was provided by open bucket twice daily. The quantity of milk given to calves was increased gradually from four litres/day on day one, to six litres/day on day 21; this amount was then maintained until day 48. Milk weaning was carried out over three days by reducing milk volume over six feeds (2.5, 2.0, 1.75, 1.5, 1.0 and 0.5 litres) from day 48, and on day 51, three litres of warm water was provided as this can reduce stress at weaning [50].

On day 55 each block of five calves were grouped together by removing the pen walls that separated them, to leave one larger pen made from the original perimeter walls. Each block of calves was then moved to a straw barn on day 60, so that every 3–5 days the group size in the barn increased by five individuals. When the *barn grouping* part of the study began, all calves were housed within a 220m^2^ section of this 1012m^2^ barn. Straw feed and pellets (BOCM, Super Rearer 18 + deccox) were delivered (into a trough) morning and evening, and water was available *ad libitum*.

### Statistical methods

Data for pair-reared calves were averaged to give one value per pen. Where presented as a percentage or proportion, data were transformed using the arcsine square-root transformation. When analysing data around weaning, means were calculated for days 45–47 (pre-weaning), 48–50 (weaning) and 51–53 (post-weaning). Where multiple tests were carried out on one dataset, false discovery rate (FDR) adjusted *p*-values were calculated. These are quoted as a *q*-value using the two-stage sharpened method [51]. Statistical analysis was conducted using IBM SPSS software vs.19 and R statistical software (R Core Team, 2014).

### Pen rearing

#### Measures of health, production and weaning stress

Health checks of individuals were carried out daily by the experimenter on days 5 to 54, according to the University of Wisconsin-Madison Health Scoring Criteria, that was developed by veterinarians to identify calves that should be treated for bovine respiratory disease [53]. Faecal scores were recorded, and cough score, nasal discharge score, eye score, and ear score were added together to give an overall respiratory score. Daily concentrate intakes (per pen) were determined on days 5 to 54 following morning milk feeding, by weighing feed remaining in the feed bucket and deducting it from the amount provided on the previous day. Vocalisations (per pen) were counted by the experimenter for one hour at approximately 8am (following morning milk feeding, on the days this was given) for three days pre-weaning, weaning and post weaning. Body weight was recorded on entry to the study and on day 55 using a weigh-scale (Iconix FX1, NZ.) and a weigh-band (developed for Holstein-Friesian heifers by the Agri-Food and Biosciences Institute, Belfast, in conjunction with the Royal Veterinary College, AFBI, 2011). An additional measurement was taken on day 47 using the weigh-band only. Specific growth rate (SGR) [54] was used to calculate weight gain, using Eq 1, where W_1_ is the weight at sample point one, W_2_ is the weight at sample point two and *t* is the time in days between sample points one and two.

$$Specific\;growth\;rate\;\left[\%\right]=100\left(\frac{\left(\text{ln}W_{2}-\text{ln}W_{1}\right)}{t}\right)$$ (1)

#### Between-treatment differences in overall health, growth and intakes

We tested for treatment differences in faecal and respiratory scores over the whole pen rearing period using a one-way MANOVA. One-way ANOVA were used to test for treatment differences in: concentrate intake over the whole trial, weight at the start of the trial, and mean SGR over the whole trial period.

#### Between-treatment differences in vocalisations, growth and concentrate intakes during weaning

Friedman’s ANOVA was used to test whether the number of vocalisations differed between the pre-weaning, weaning and post-weaning three-day periods. Kruskal-Wallis tests were used to assess for any significant differences in the number of vocalisations between treatments during each period. ANOVA were performed to test for treatment differences in: SGR from days 47–55 (to assess the effect of weaning), concentrate intake during the pre-weaning, weaning and post-weaning three-day periods, and to test whether any differences found were dependent on weaning stage.

### Barn grouping

#### Spatial proximity loggers

In order to measure social associations between the calves remotely and continuously, spatial proximity loggers (model E2C181C) made by Sirtrack Ltd. (New Zealand) were deployed on day 55. These devices are attached to collars that are worn around the animal’s neck, and give users information on frequency and duration of close proximity between individuals. They function by both broadcasting unique identification codes over an ultra-high frequency (UHF) channel, and searching for the ID codes of others. When loggers enter a pre-determined distance range (set by the user via alteration of the power setting of a UHF coefficient range), both record the encountered logger’s ID, the date, the start and end time of the encounter, and its duration. Users can also determine the duration that loggers need to be out of contact range for an encounter to terminate (the “separation time”) prior to deployment. Here, proximity loggers were set to a UHF value of 45 with a separation time of 120s, which equated to detecting contact between calves when they were within 1.5m (approximately) of each other.

Data were downloaded from proximity loggers and prepared for analysis using the R packages ‘Matrix’ [55] and ‘chron’ [56]. Four week-long association matrices were constructed separately for weeks 1–4 by summing the duration of all associations between each dyad during each week. All 1-second contact records were omitted from the analysis, as these are not deemed reliable [57, 58]. Data were corrected according to methods from Boyland et al., [59], to account for the sampling bias that can arise when loggers vary in their performance. This method involves first measuring the percentage difference in association durations (e.g. the percentage difference between the total time logger A recorded contact with logger B, and the total time logger B recorded logger A) between all logger dyads, then reducing the association durations for each logger according to its logging bias with the most under-recorded logger. For example, if logger A had a logging bias of 5% when compared to the most under-recorded logger, the duration that logger A recorded contact with all other loggers would be reduced by 5%. This allows us to standardise associations between loggers relative to each other (please see Boyland et al., [59] for a full description of the method).

#### Network stability

We examined the stability of social associations at the group level, during the month that all calves were grouped together in the barn. The four week-long association matrices were compared with each other using the ‘mantel’ function (method = Spearman’s rank correlation) of the ‘vegan’ package [60] in R. A mantel test determines the correlation between two dissimilarity matrices; the significance of which is then assessed using a node-label permutation known as the quadratic assignment procedure(n = 4999).

#### Social differentiation

We calculated social differentiation (the heterogeneity of associations at the group level) in each of the four week-long networks, to determine whether associations between calves were more varied than would be expected given a null hypothesis that individuals associate uniformly. The statistic was adapted from Whitehead [61] appendix 9.4, calculated using Eq 2 in which the difference between the observed (duration of associations) value and the expected (duration of associations if all individuals associated uniformly) value is summed for each dyad, and then divided by the total number of dyads.

$$S=\frac{\sum_{i}^{N}\sum_{j}^{N}{\left(O_{ij}-E_{ij}\right)}^{2}}{N\left(N-1\right)}$$ (2)

#### Assortment

To test if pen rearing affected patterns of social association during barn grouping, we tested for assortment by treatment and familiarity in week one and week four after regrouping. We used a Markov Chain Monte Carlo (MCMC) framework to measure the relationship between the dependent variable, association strength, and the fixed factors (familiarity and treatment). Familiarity was measured for each calf dyad as the number of days that they had been in full social contact before the first day of barn grouping; therefore familiarity between individuals ranged from 0 days (i.e. there was no prior full social contact between calves in block eight and calves in blocks one to seven) to 76 days (i.e. calves that were paired at five days old in block one were most familiar with each other). To test for assortment by treatment, dyads were awarded a ‘0’ if they were of the same treatment and a ‘1’ if they were of different treatment. Calf ID was included as a random effect in all models. The undirected nature of association measures (all calves act as individual A and B of a dyad in the dataset) are accounted for within the ‘MCMCglmm’ package [62]. To satisfy assumptions of normality, we log-transformed the dependent variable, association strength. As our networks were completely saturated (all calves interacted), we have made the assumption that typical dependencies generated by heterogeneous network structure, such as transitivity (if *A* and *B* are connected and *B* and *C* are connected, then there is a greater chance of *A* and *C* being connected), will not affect our analysis. Other than variation in association strength (the dependent variable), the underlying social environment in this study was homogenous, with the potential for structural dependencies which are commonly found to influence the structure of social groups (such as aforementioned transitivity) being limited (see Snijders [63] for further reading on this topic). As such, we chose to model association strength in terms of treatment and familiarity using a Bayesian approach. We ran the MCMCglmm models with all possible combinations of fixed factors (familiarity and treatment) and identified the best fitting model as that with the lowest deviance information criterion (DIC) [64].

#### Between-treatment differences in coefficient of variation (CV) in association strength

We calculated the CV in total association strength for calves in week one and week four. ANOVA (with 5000 bootstrap permutations, used to calculate confidence intervals and p values) were then used to test for significant differences in CV in association strength between calves of different treatments. Additionally, we re-ran the tests after omitting contact durations between calves that were pair-reared together, in order to determine whether there were treatment differences CV in association strength, independent of the bond between individuals that were paired during pen rearing.

#### Between-treatment differences in percentage of time spent with ex-penmate

We calculated the percentage of social association time that individuals spent with the calf they were paired with during pen rearing, and tested for differences between *P5* and *P28* calves in week one and week four, using a one-way ANOVA.

#### Between-treatment differences in social network measures

Weighted degree centrality and eigenvector centrality scores were calculated for each calf, in UCINET 6 [65], for week one and week four of barn grouping. However these measures were highly correlated (Spearman’s rho = 0.992 (week 1) and 0.997 (week four), significant to <0.0001), therefore we only used weighted degree centrality in the analyses. This measure is the sum of the strength of an individual’s associations. We ran one-way ANOVA (with 5000 bootstrap permutations, used to calculate confidence intervals and p values) to test for differences between *I*, *P5* and *P28* calves in weighted degree centrality in both week one and week four.

### Ethical considerations

This research was mainly observational and non-invasive. Ethical considerations of this study were evaluated according to guidelines produced by the Association for the Study of Animal Behaviour (2012) and the research was approved by the Department of Psychology’s ethical review group at the University of Exeter.

## Results

### Pen rearing

#### Overall health, growth and intakes

Calves weighed, on average 37.39 (±5.55)kg on arrival at the rearing unit and there was no significant difference in arrival weights between the treatments (*I*: 35.11 (±7.06)kg, *P5*: 39.39 (±3.59)kg, *P28*: 36.39 (±6.11)kg, *F*(_2, 36_) = 1.99, *p* = 0.152). One calf, allocated to treatment *I*, died on day 12, and the cause of death was unknown. All data for this calf was omitted from the analysis. The mean (±SD) percentage of days with a faecal score above one (indicating impaired health) was 5.25 (±2.71)%, and a respiratory score above zero (indicating impaired health) was 5.00 (±4.32)%. There was no significant effect of treatment on health scores (MANOVA: *F* = (_4, 40_) = 0.56, *p* = 0.69 (Pillai’s Trace); mean (±SD) respiratory statistics *I*: 4.57 (±6.5), *P5*: 4.88 (±2.10), *P28*: 5.5 (±4.17); mean (±SD) faecal statistics *I*: 5.62(±3.28), *P5*: 5.52(±2.28), *P28*: 4.68(±2.84)).

The mean concentrate intake over the whole trial period was 448.41 (±171.43)g/day, and there were no significant treatments differences in concentrate intake over the whole trial (*I*: 425.17 (±192.63)g, *P5*: 536.55 (±175.29)g, *P28*: 380.61 (±123.11)g; ANOVA: *F*(_2, 20_) = 1.89, *p* = 0.177). There was also no significant difference in growth over the entire study period between the treatments (ANOVA: *F*(_2, 20_) = 0.70, *p* = 0.510). The mean specific growth rate across the whole trial period was 1.06(± 0.14)%.

#### Vocalisations around weaning

**Fig 1:**
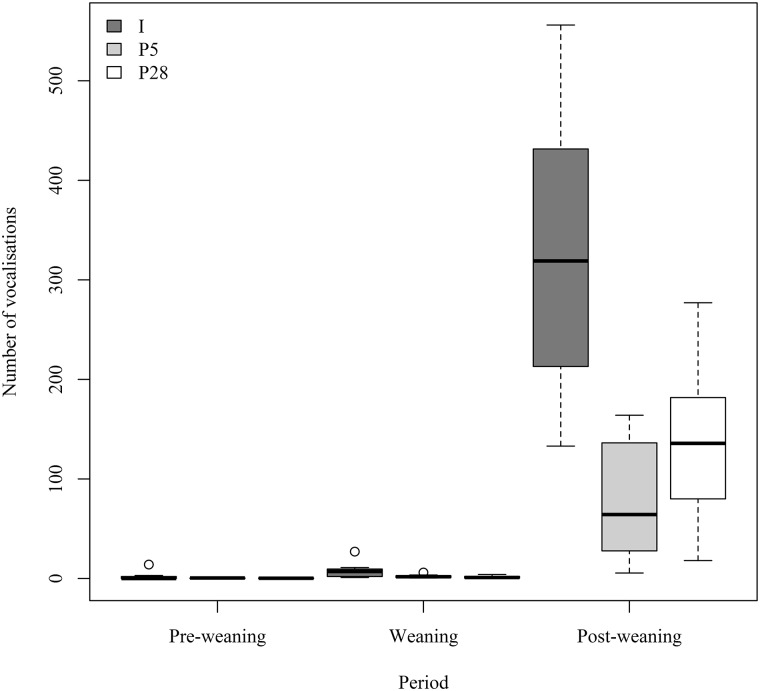
Vocal responses of calves to weaning. The total number of vocalisations, during one hour observations of calves, over each three-day period (pre-weaning, weaning and post-weaning).

The number of vocalisations was significantly affected by stage of weaning (Friedman’s ANOVA: χ^2^ (2) = 41.42, *p*< .001). Calves vocalised significantly more during the weaning period (1.25 ±1.93 calls/h) than during the pre-weaning period (0.34 ±0.97 calls/h; Wilcoxon: *Z* = -3.180, *p* = 0.001, *q* = 0.001) and significantly more during the post-weaning period compared to the weaning period (*Z* = -4.197, *p* <0.001, *q* <0.001). There was no significant difference in the number of vocalisations between treatments during the pre-weaning period (*I*: 0.86 ±1.72 calls/h, *P5*: 0.10 ±0.9 calls/h, *P28*: 0.10 ±0.13 calls/h; Kruskal-Wallis: *H*(2) = 0.19, *p* = .701; Fig 1). However, treatment had a significant effect on the number of vocalisations during the weaning period (*I*: 2.76 ± 1.14 calls/h, *P5*: 0.73 ± 0.21 calls/h, *P28*: 0.46 ±0.16 calls/h; Kruskal-Wallis: *H*(2) = 6.46, *p* = .008; Fig 1) and post-weaning period (*I*: 109.38 ±51.40 calls/h, *P5*: 26.08 ±20.16 calls/h; *P28*: 45.42 ±26.77 calls/h; Kruskal-Wallis: *H*(2) = 11.44, *p* < .001; Fig 1). *I* calves vocalised four times more than *P5* calves during the post-weaning period (Mann-Whitney: *U* = 2.00, *p* = 0.001, *q* = 0.001) and over twice as much as *P28* calves (*U* = 7.00, *p* = .014, *q* = .007). During the post-weaning period, *P28* calves vocalised significantly more than *P5* calves (*U* = 17.50, *p* = 0.137, *q* = 0.048).

### Barn grouping

#### Network stability

All week-long association matrices were significantly positively correlated, indicating a degree of stability in the calves’ network (Table 1). The R squared value for the correlation between weeks one and four suggests around 50% of the network was stable from the start to the end of the month.

**Table 1:** Stability of the calf social network during barn grouping.

Weeks	R^2^	*p* value
**1, 2**	.572	0.0002
**1, 3**	.527	0.0002
**1, 4**	.505	0.0002
**2, 3**	.576	0.0002
**2, 4**	.554	0.0002
**3, 4**	.637	0.0002

Correlations between each week-long matrix, measuring network stability across weeks 1–4. Significance values were generated by comparing the observed values with those from 4999 null networks.

#### Social differentiation

In each week—long social network there was significant social differentiation (Table 2) which demonstrates that calves associated non-uniformly, spending more or less time with other individuals than would be expected by chance.

**Table 2:** Social heterogeneity of calves, measured at the group level, during barn grouping.

Network	Social differentiation		95% quantile of null distribution	*p* value
	Observed	Median of Nulls		
**Week 1**	20187018	606831.1	651672.5	0.0002
**Week 2**	10949424	453363.6	489180	0.0002
**Week 3**	10563239	516759.3	555467.9	0.0002
**Week 4**	36357611	1005410	1085073	0.0002

There was significant social differentiation for each week—long social network; calves spent more or less time associating with other individuals than would be expected by chance.

#### Assortment

There was significant assortment by familiarity in week one (posterior mean = 1.417, p<0.001, DIC = 4490.49) and week four (post. mean = 1.037, p<0.001, DIC = 4664.901); calves spent more time with those they were more familiar with (in terms of duration of full social contact). Calves were not significantly assorted by treatment in week one (post. mean = -2.97, p = 0.076, DIC = 4876.727) or week four (post. Mean = -2.124, p = 0.2, DIC = 4876.406). The posterior mean refers to the mean of the posterior distribution, as generated by the Bayesian model for the given parameter and is indicative of effect size.

#### Between-treatment differences in coefficient of variation (CV) in association strength

**Fig 2:**
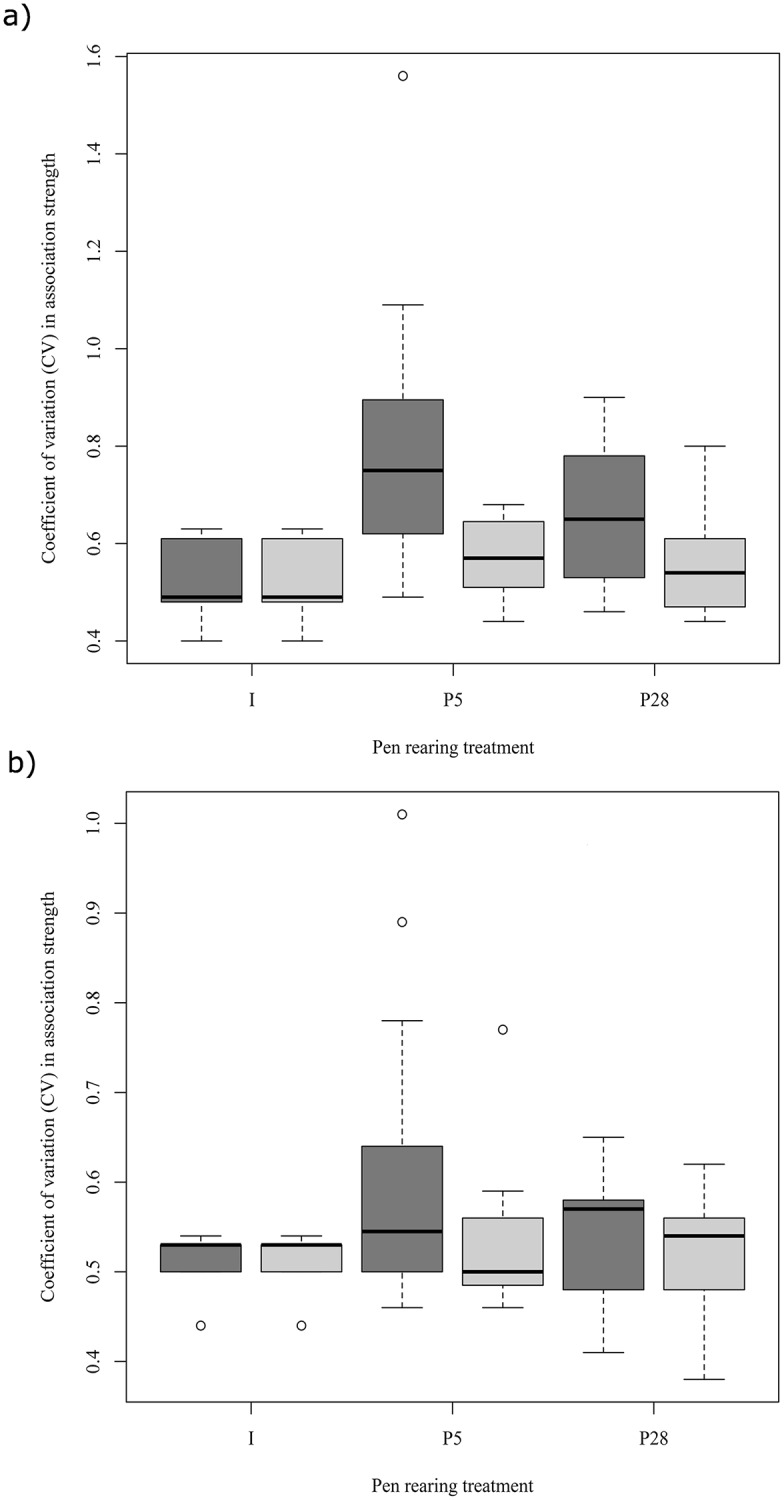
Heterogeneity of social interactions. The coefficient of variation (CV) in association strength for calves in each rearing treatment during week one (a) and week four (b). Dark grey boxes show all data; light grey boxes show data when associations between previously paired calves was omitted.

There was a significant difference in CV in association strength between treatments during week one (F(_2,34_) = 5.238, p = 0.011; Fig 2a). A Bonferroni post hoc test revealed that *P5* calves had significantly higher CV in association strength than *I* calves. However, there were no significant differences in CV in association strength between treatments during week four (F(_2,34_) = 1.883, p = 0.169; Fig 2b). After omitting the duration of time that calves spent with their ex-penmate from the analyses, there were no significant differences in CV in association strength between treatments in week one(F(_2, 34_) = 0.504, p = 0.609; Fig 2a) or week four (F(_2, 34_) = 0.274, p = 0.762; Fig 2b).

#### Between-treatment differences in percentage of time spent with ex-penmate

The percentage of social association time individuals spent with the calf they were paired with during pen rearing (referred to as ‘percentage pair-time’) was not significantly different for *P5* and *P28* calves in week one(F(_1,24_) = 0.831, p = 0.371) or in week four (F(_1,24_) = 0.583, p = 0.453). Overall there was a significant decrease in percentage pair-time from week one (11.015±6.049) to week four (6.827±3.95) (paired samples t-test; t(25) = 7.2. p<0.001).

#### Between-treatment differences in social network measures

There were no significant differences between treatments in the weighted degree centrality of calves in week one (F(_2,34_) = 2.402, p = 0.107) or in week four (F(_2,34_) = 0.763, p = 0.475).

## Discussion

This study investigated the effects of social contact during early rearing on the health, production and welfare of dairy calves, on a UK commercial farm. Individual and paired pen rearing were compared and the effect of age at pairing was explored, with particular interest in how this influenced the stress buffering effect of social support at weaning. Following pen rearing, calves were grouped and moved to a barn, and the social network that formed was measured using data from spatial proximity collars.

Dairy farmers often avoid grouping calves in early life due to the anticipated negative consequences for health and production. However, in the current study, there was no evidence of health or production differences between calves that were housed in pairs or in individual pens. As required by EU legislation (Council Directive 97/2/EC), individually reared calves were still able to make direct (oral and nasal) contact through the slots in the partitions between pens, therefore there was still potential for direct pathogen transmission between individually reared calves. A higher incidence of diarrhoea and respiratory diseases for group-reared calves has been reported (e.g. Maatje, Verhoeff (20)), though the difficulties of early disease detection in larger groups [66] may contribute to such findings. It is believed however, that calf immunity, good hygiene, ventilation and adequate feeding can have a greater impact on susceptibility to disease than housing type [23].

Pair rearing or age at pairing did not influence the concentrate intake or weight gain of calves during pen rearing in this study. Previous studies have described diverse findings. For example Maatje, Verhoeff (20) observed reduced feed intake and lower weight gain in group-reared calves and suggested this was due to competition for feed. De Paula Vieira, Von Keyserlingk (38) and Warnick, Arave (48) found significantly higher concentrate intake for group-reared calves but reported no significant increases in weight gain. In contrast, studies by Bernal-Rigoli, Allen (67), Costa, Meagher (68), Jensen, Duve (69) and Tapki (70) found that group reared calves had significantly higher feed intake and weight gain. In this study, weight gain over the whole trial period did not appear influenced by pair-rearing or by age of pairing, comparable to studies by Arave, Mickelsen (71), Broom and Leaver (47), De Paula Vieira, Von Keyserlingk (38), Duve, Weary (72). Experimental methodology or management practices may account for these variations.

Vocalisations are a common behavioural response to the stress of milk withdrawal from calves [50]. Therefore unsurprisingly, we found a significant increase in the number of vocalisations during weaning and in the days post-weaning. The greatest effect was seen during the post-weaning period, when individually reared calves vocalised significantly more than pair-reared calves. In addition to the presence of a conspecific, the efficacy of social buffering may be influenced by several factors, including the strength of the affiliation between the individuals [73]. Indeed in the current study, the significantly lower number of vocalisations exhibited by *P5* calves compared to *P28* calves suggests a greater affiliation between those paired at an earlier age and, resultantly, more effective social buffering. There is often a growth check in calves at weaning [34], and in this study growth rates over the weaning period were indeed lower than the average across the whole trial period. Growth rate was highest in the *P5* calves, however it was not significantly different to that of *P28* or *I* calves. Chua, Coenen (23) reported lower growth rates in individually-housed calves when compared to pair-housed calves during the weaning period. The disparity between our findings and the findings of Chua, Coenen (23) may be due to the increased concentrate intake by pair-housed calves which was not observed in this study. Social facilitation of concentrate intake, and the effects of milk allowance, have been investigated by Jensen, Duve (69). Interestingly, although calves fed an enhanced milk allowance consumed less concentrate than those given a standard milk allowance, pair housing stimulated concentrate intake in these calves (and also led to greater body weights), suggesting that pair housing and enhanced milk feeding should be used together. This provides evidence for social facilitation and further highlights the importance of social housing for calf welfare.

After calves were grouped in the straw barn, we quantified their social relationships and network structure by measuring the time they spent in close proximity to each other. We found significant positive correlations between all week-long matrices, indicating that over the one month data collection period calves were stable, to some degree, in their social associations. Correlations indicated a very similar level of consistency from weeks 1–2 and weeks 1–4. Therefore, network stability did not appear to lessen over this time. We also found that calves were socially differentiated; there was heterogeneity in the connections between individuals. In combination, these results suggest that there was stable inter-individual variation in social associations, and thus social preferences of calves were detected. In a study by Koene and Ipema (74), calf social networks were created from nearest neighbour data but the authors found no evidence that calves had preferred partners or that social relationships were stable; daily matrices were not significantly correlated (apart from oneout of 12 matrix pairs which were negatively correlated). Disparity between our results and those of Koene and Ipema (74) may be due to differences in methodology; they used a smaller group (n = 10) of older (3–4 month old) calves, compared networks on a finer time scale. They do not provide information on the history or familiarity of the calves studied.

In the current study, calf networks were assorted by prior familiarity (number of days of full social contact prior to grouping in the barn) however there was no evidence for assortment by treatment. Assortative mixing refers to the tendency for individuals to associate more with those that are similar to them in some way, and has been found in networks of adult cattle [75]. The degree of familiarity is likely to have reflected the strength of social bonds, therefore our results support previous findings of cattle associations (e.g. Raussi, Niskanen (76), Sato, Wood-Gush (31), Færevik, Jensen (77), Færevik, Andersen (78)). Assorting with familiar conspecifics can provide adaptive benefits, such as the use of information from prior experiences to improve group activities, and reduction of conflict via predetermined dominance relationships [79]. In the farm environment, familiarity may also be particularly significant in terms of improving social support. The enhanced social buffering observed in *P5* calves at weaning, along with the preference of calves for familiars when grouped in the barn, supports other studies (e.g. Rault (29), Bøe and Færevik (43), Raussi, Niskanen (76)) suggesting that human-managed animals should be encouraged to form stable social bonds with conspecifics over time, due to the welfare benefits.

Although familiarity between individuals may vary considerably when a group first forms, over time the relative variation decreases, suggesting the effect of initial familiarity on social bond strength would eventually become insignificant. However research indicates that early preferences persist into adulthood, which may indicate a sensitive period for social bond formation in cattle [76]. Vitale, Tenucci (32) observed calves in a free-ranging context, and found that time associating with peers was highest from 11 to 40 days of age. Indeed a number of studies show that early social relationships remain for substantial time periods (e.g. Sato, Wood-Gush (31), Reinhardt and Reinhardt (80), Bouissou and Hövels (49), Bouissou and Andrieu (81), Raussi, Niskanen (76)). For example, Bouissou and Andrieu (81) found that heifers grouped at birth formed more preferential associations than those grouped at six or 12 months, and in a study by Gygax, Neisen (82) cattle that had been reared together were more synchronised. In the current study, both pair-housed treatments involved full social contact for calves within this potential sensitive period. Although there was an important distinction between these treatments during weaning (*P5* calves had a lower stress response to weaning), in the month following regrouping there were no substantial differences in the amount of time calf pairs spent in close association. In week 1, the CV in association strength between calves was significantly higher for *P5* calves than *I* calves, indicating that their (*P5* calves’) associations were more heterogeneous. This effect appears to have been driven by the time *P5* calves spent with their ex-penmates, as there were no significant differences when these associations were omitted. Further, by week 4 these treatment differences in association patterns were no longer significant. Research into the effect of heterogeneous social associations on disease transmission in groups of dairy calves would be an interesting avenue for future study.

The early social environment has been shown to affect calves’ development in previous studies. For example individually housed calves were more reactive to environmental and social novelty (indicated by increased rates of defecation, kicking and vocalisations) than pair-housed calves [83] and performed significantly worse in cognitive tasks [40]. Social performance later in life can also be affected; group-housed calves achieved higher dominance rank than individually reared calves [46, 48]. Recently, developmental conditions have also been linked to social network position [13], which can have consequences for access to resources (e.g. Aplin, Farine (84)) and exposure to pathogens (e.g. Bull, Godfrey (85)) etc. In this study there were no significant differences based on pen rearing treatments in weighted degree centrality, suggesting that the level of prior social contact did not affect calves’ position in the social group at this age. However further work is required to explore this fully; this research was carried out on a commercial farm, where management determined that calves entered the barn, by block, on different days (five calves entered the group every three days). As we only measured networks after all calves had entered the group, we may have missed initial treatment differences in social behaviour, particularly as *I* calves were experiencing full social contact for the first time. Similarly, we only quantified the social network structure for one month, and differences in network position may appear later in life, when social behaviour develops further and dominance hierarchies form. Future work should be aimed at understanding the temporal dynamics of dairy cattle from early rearing through to lactation and beyond.

### Conclusions

The present study could not detect any health or production effects of calf rearing treatment, but paired calves showed a reduced vocal response to weaning which indicates a stress buffering effect. Calves paired at 28 days conferred noticeable social buffering effects to each other at weaning, however more effective social buffering was achieved by pairing calves at five days old. Allowing full social contact with other calves as early as possible should therefore be encouraged. The calf social network structure following barn grouping demonstrated stability in the associations between individuals, with relationships reflecting prior familiarity. This study suggests that cattle begin forming important social preferences during the first few months of life; understanding social relationships and group structure is essential for minimising stress and improving welfare in the dairy industry.
